# Comparing the Ecological Impacts of Wind and Oil & Gas Development: A Landscape Scale Assessment

**DOI:** 10.1371/journal.pone.0081391

**Published:** 2013-11-27

**Authors:** Nathan F. Jones, Liba Pejchar

**Affiliations:** Department of Fish, Wildlife and Conservation Biology, Colorado State University, Fort Collins, Colorado, United States of America; University of Kansas, United States of America

## Abstract

Energy production in the United States is in transition as the demand for clean and domestic power increases. Wind energy offers the benefit of reduced emissions, yet, like oil and natural gas, it also contributes to energy sprawl. We used a diverse set of indicators to quantify the ecological impacts of oil, natural gas, and wind energy development in Colorado and Wyoming. Aerial imagery was supplemented with empirical data to estimate habitat loss, fragmentation, potential for wildlife mortality, susceptibility to invasion, biomass carbon lost, and water resources. To quantify these impacts we digitized the land-use footprint within 375 plots, stratified by energy type. We quantified the change in impacts per unit area and per unit energy produced, compared wind energy to oil and gas, and compared landscapes with and without energy development. We found substantial differences in impacts between energy types for most indicators, although the magnitude and direction of the differences varied. Oil and gas generally resulted in greater impacts per unit area but fewer impacts per unit energy compared with wind. Biologically important and policy-relevant outcomes of this study include: 1) regardless of energy type, underlying land-use matters and development in already disturbed areas resulted in fewer total impacts; 2) the number and source of potential mortality varied between energy types, however, the lack of robust mortality data limits our ability to use this information to estimate and mitigate impacts; and 3) per unit energy produced, oil and gas extraction was less impactful on an annual basis but is likely to have a much larger cumulative footprint than wind energy over time. This rapid evaluation of landscape-scale energy development impacts could be replicated in other regions, and our specific findings can help meet the challenge of balancing land conservation with society’s demand for energy.

## Introduction

Global changes in energy production are occurring as a result of increased demand for clean, cheap, and domestic power coupled with rising consumption and a finite supply of fossil fuels. Wind energy is at the forefront of this transformation and is now the world’s fastest growing source of electricity [1]. This trend is driven in part by goals such as the U.S. Department of Energy’s (DOE) intent to achieve 20% of electrical power from wind by the year 2030 [2]. The benefits of wind energy include low lifecycle emissions of greenhouse gases [3], which support the perception that wind is a ‘clean’ alternative to fossil fuels such as oil and natural gas. However, focusing on emissions alone ignores the impacts of ‘energy sprawl’, or the increasing amount of land altered for energy production [4]. The land-use required for energy production is predicted to grow rapidly with human population growth [4]. However, existing estimates are variable and highly dependent on evolving technologies [4]–[6]. The degree to which wind energy and traditional sources of energy (e.g. oil and natural gas) result in negative impacts to biodiversity and ecosystem services is not well understood. Empirical research on the impacts of energy sprawl on biodiversity and ecosystem services is scarce, inconsistent, and unevenly distributed among energy types and faunal groups [7]. Most literature on the impacts of wind development focuses on avian and bat collisions with wind turbines [7], [8]. Literature on the impacts of oil and gas development in western North America has focused largely on habitat degradation impacts to only a few species of concern: primarily sagebrush or grassland obligates and ungulates [9]–[11]. The impacts of energy development on other important characteristics of the natural and built landscape, such as invasive species, carbon storage and sequestration, and water resources are of great concern to society, but have received very little attention in the literature.

Measuring the impacts of energy development on natural communities is traditionally accomplished through field studies, but this research is arduous, expensive, and sometimes impractical at the scales or time frames that matter for decision making. Landscape-scale assessments can be used to complement field research and to evaluate impacts on the spatial and temporal scale needed to guide land-use planning and management decisions. The use of indicators as surrogates for biodiversity measurements have proven effective in this context [12], and aerial imagery and geospatial data can be used to remotely quantify these indicators over large areas and through time [13].

The following indicators (Table 1), which have been shown to affect biodiversity, can be directly or indirectly measured from aerial imagery and used to assess net conservation impacts [12]. Habitat loss and fragmentation are generally regarded as the leading causes of biodiversity loss [14], [15] and are easily digitized and quantified from aerial imagery. Wildlife mortality resulting from collision or contamination due to various sources (e.g. turbines, vehicles, power lines, reserve pits) impacts local populations and may also have community and ecosystem level effects [16]. Alien species are the second greatest agent of species endangerment [17], and although the presence and extent of invasive plant cover is difficult to assess on a landscape scale, the extent of invasion potential can be estimated based on the amount of human activity and disturbance [18]. Finally, it is important to understand the impacts to ecosystem services such as biomass carbon stock and water resources from energy development in light of climate change and diminishing supplies of freshwater. Geospatial estimates of biomass carbon stock are readily available [19], and water consumption and loss can be roughly estimated from energy infrastructure and the extent of impervious surfaces [20].

**Table 1:** List of indicators and the measures/metrics used to quantify impacts.

Indicators	Measures/Metrics
1. Direct Habitat Loss	Total hectares of direct, permanent or temporary habitat loss
2. Fragmentation	GISFrag: mean Euclidean distance to habitat loss
3. Potential Mortality	Total number of turbines, towers, evaporation ponds, and reserve pits. Length of roads and power lines.
4a. Susceptibility to Invasion	Total meters of linear features
4b. Susceptibility to Invasion	Total hectares of temporary disturbance
5. Carbon Sequestration	Total tons of biomass carbon lost
6a. Water Resources	Total gallons of water consumed per year
6b. Water Resources	Total hectares of impervious surface

The indicators listed above (and in Table 1) act as surrogates for biodiversity and ecosystem services based on the assumption that, for instance, an increase in mortality, habitat loss, or fragmentation reduces biodiversity [15], [16], [21] and a decline in carbon storage potential and water quality and quantity affects the ability of a region to provide ecosystem services to human communities [22], [23]. An advantage of this approach is that indicators can be selected based on the strength of ecological principles and existing spatial data in ways that build on previous studies but retain the flexibility to be refined over time [24], [25]. The drawback of this approach is that it assumes impacts to biodiversity and ecosystem services in a particular location based on generalized findings from the literature that may be more or less relevant to the study area. Additionally, because the impacts are based on surrogates of biodiversity and ecosystem services, this approach cannot produce conclusive evidence of energy impacts on any single species. Despite this limitation, the indicators in this study are particularly well suited for evaluating the nature and extent of energy sprawl because they respond directly to changes in land-use and these changes are detectable immediately, whereas changes in species richness and abundance may display significant lag times [12], [26].

This study combines the use of aerial imagery, geospatial data, and the indicators of biodiversity and ecosystem services described above to rapidly assess impacts from energy development. Our objectives are to compare the net impacts of oil and gas to wind energy development on a per unit area and per unit energy basis, and to compare the impacts of both energy types to other land-uses. Although we recognize that the energy portfolio for this region and others is likely to employ an “all of the above” approach, this comparison of ecological impacts will help policy makers reach better-informed decisions on how to invest in and plan for energy in a region. We also emphasize that there are challenges inherent in making a direct comparison between oil and gas and wind because electricity is produced directly from wind turbines but oil and gas requires additional processing to generate power. In contrast to a life-cycle analysis, which generally includes transportation, transmission, and energy conversion, this study specifically responds to emerging concerns over the land-based impacts of energy sprawl and thus is deliberately focused on the farms and fields where production occurs.

## Methods

### Study Area

The study area was defined as the political boundaries of Colorado and Wyoming, U.S.A. These two states were chosen because they exemplify areas with substantial historic, current, and potential future wind, oil and natural gas development. The locations of all existing wind turbines in Colorado and Wyoming were obtained from the U.S. Geological Survey [27], [28]. Energy developers supplemented this data to ensure a complete census as of 11 September 2011. The locations of all current and historic oil and natural gas wells (conventional and unconventional) were downloaded from the Colorado and Wyoming Oil and Gas Conservation Commissions (COGCC and WOGCC) [29], [30]. The point locations of turbines and wells were buffered by 500 m to create three separate and spatially distinct polygonal features representing 1) wind energy, 2) oil and natural gas, and 3) all remaining areas without energy development, denoted as the ‘reference’ stratum.

### Sampling Design and Data Collection

**Figure 1:**
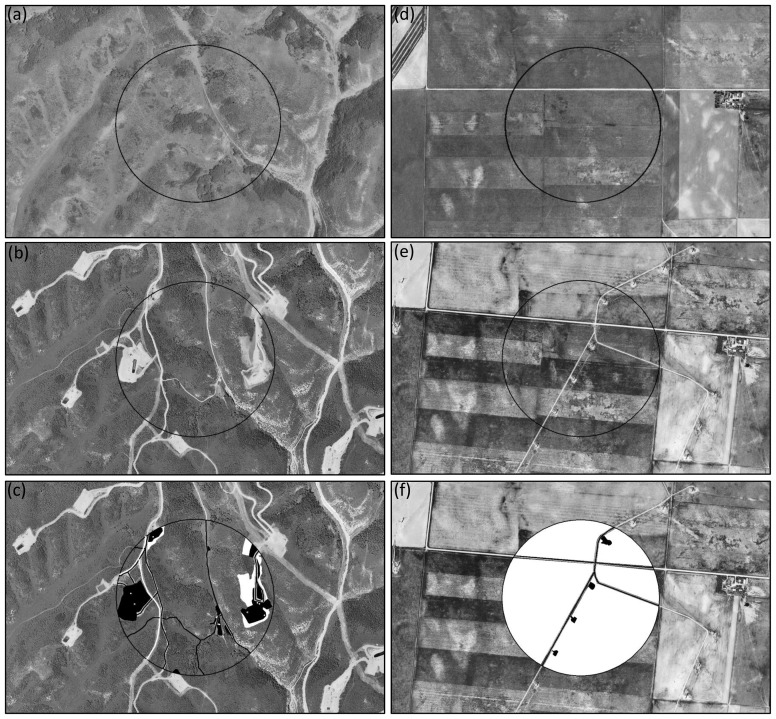
Examples of sample plots used to quantify impacts to indicators of biodiversity and ecosystem services. Western Colorado landscape before (a) and after (b) natural gas development. Eastern Colorado landscape before (d) and after (e) wind energy development. Sample plots with habitat loss digitized (c,f) as impervious (white) and non-impervious (black). Imagery from the National Agriculture Imagery Program (USDA Farm Service Agency) and National Aerial Photography Program (USGS) are representative of the imagery used during data collection.

**Figure 2:**
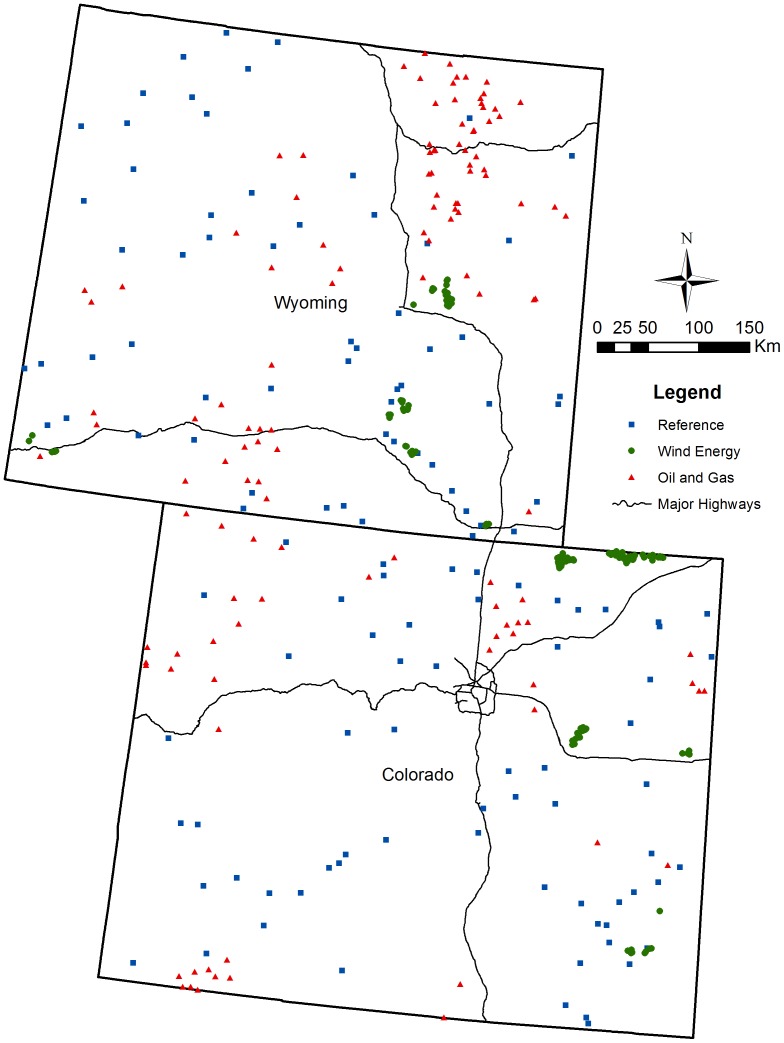
Study area and sample plots. The study area is defined by the political boundaries of Colorado and Wyoming and includes 375 randomly selected, 1-km diameter sample plots, stratified by wind energy, oil and gas, and other/underlying land-uses (reference stratum).

A total of 375 stratified (125 per strata), spatially balanced, simple-random 1-km diameter plots were selected from within the three feature classes (Figure 1 and Figure 2). The scale of our plots is consistent with previous landscape scale studies conducted in the region [13]. Within each sample plot we digitized the human “footprint” (i.e., any area directly affected by human activity) based on imagery from the National Agriculture Imagery Program (NAIP) supplemented with Google Earth™ conglomerate imagery packages to identify changes over time. Only one author (NFJ) digitized and/or proofed all features to maintain consistency in data collection. In spring 2012 we field mapped any energy infrastructure that was constructed since the most recently available imagery. The boundaries of areal features were digitized as polygons and linear features as polylines. Point features were used to denote meteorological towers, reserve pits, evaporation ponds, turbines and wells. Each digitized feature was classified by land-use type (i.e., wind, oil and gas, agriculture, residential, etc.) based on a classification scheme modified from Leinwand et al. [13]. Features that could be attributed to energy development were digitized as an energy feature, and features that were separate from or pre-dated energy development were attributed to one of several underlying land-uses. These data enabled us to quantify the proportional change and the net impact of energy development. Each polygon and polyline was classified using Land Based Classification System feature types [31]. Agricultural fields and croplands were considered “loss of habitat” and were therefore digitized, whereas pastures and rangeland were not. Water bodies created by dams or other anthropogenic barriers were classified as a human footprint, whereas natural water bodies were not.

### Measuring Impacts to Indicators

To quantify impacts to biodiversity and ecosystem services, a set of six unique indicators (Table 1) were selected as surrogates for empirical measurements. Habitat loss was calculated as the total area either temporarily or permanently affected by human activity within each plot due to wind energy, oil and gas, and underlying land-uses. To quantify the degree of fragmentation within each plot, we used the GISFrag metric which is equivalent to the mean distance to the nearest habitat loss within a given area [32]. We first digitized all habitat loss within each plot and within 500 m of each plot to account for disturbances located immediately outside of the sample plot. We then created a grid of 30 m cells with values equal to the Euclidean distance to the nearest habitat loss. The mean value of all cells in the sample plot was used to measure fragmentation. A 500 m buffer was chosen because it is a common distance within which many species are affected by disturbance [10], [11], [33], [34].

Unique sources of wildlife mortality from wind energy include turbines and meteorological towers [35]. Sources of mortality limited to oil and natural gas fields include evaporation ponds and reserve pits [36], which are both designed to store and/or separate the liquid and solid byproducts of drilling. Evaporation ponds are generally defined as larger and more permanent facilities than reserve pits which are smaller and only present during active drilling. Evaporation ponds were identified from aerial imagery, however, reserve pits are usually removed and the landscape reclaimed shortly after drilling, making reliable identification of reserve pits from imagery difficult. Information on whether reserve pits were used at particular wells can sometimes be found in COGCC and WOGCC databases. Where this information was not available, we assumed a reserve pit was present at each well because until recently these features were almost ubiquitous with oil and gas wells in Colorado and Wyoming (S. Ellsworth, personal communication). Reserve pits are now quickly becoming obsolete as many regulators and operators are adopting closed-loop systems which eliminate the need for open pits.

We also calculated the total length of roads and power lines within each plot, both before and after energy development. Potential for wildlife-vehicle collisions was assumed to be positively related to the length of road within each plot. Because the amount and type of vehicle activity is highly variable and dependent on numerous factors, we assumed vehicle activity was approximately equal in both wind and oil and gas developments.

To assess the potential for the introduction and establishment of invasive species we quantified two sub-indicators. First, we measured the total length of all linear features (i.e., roads, power lines, buried pipelines) to represent the relative amount of human activity [37] and edge area [38] within each plot. Second, we quantified the area of visible temporary (i.e., non-impervious, non-cropland) disturbance within each plot. These values represent areas of disturbance where invasion has a higher probability of occurring [39].

Biomass carbon stock values were estimated from data provided by Ruesch and Gibbs [19] which is derived from International Panel on Climate Change [40] methods. We matched vegetation types listed in Ruesch and Gibbs [19] to National Land Cover Dataset land cover types. The total area of each land cover type was reported for each plot as well as each digitized impervious feature. This information was used to calculate the total biomass carbon stock in each plot, as well as the total biomass carbon stock lost due to impervious surfaces.

To assess the relative impacts to water resources, the magnitude of annual water loss and area converted to impervious surfaces were estimated for each sample plot. Because water usage per well is either not reported or is proprietary data, this value was estimated based on publicly available information [41], [42]. The total area of impervious surfaces, as determined from aerial imagery, was calculated within each plot and was equivalent to permanent habitat loss.

We also compared impacts per unit energy consumed by an average American in one year (i.e., 317 million British thermal units [MMBtu])[43] as a means of demonstrating potential per capita impacts within a single year of production and over the lifespan of each type of energy development in a particular location. Production data for each wind energy facility since 2001 is available from the Energy Information Administration [44] and production data for each oil and gas well is available from the COGCC [29] and WOGCC [30] since 1999 and 1973, respectively. Mean annual production per turbine (annual facility production divided by number of turbines) and per well was quantified from the available data and summed to calculate total annual production per plot. These values were then divided by 317 MMBtu, to calculate the units of energy per plot that would support an average American in one year. Finally, for wind and oil and gas separately, the mean impact of each indicator per plot was divided by the mean units of energy (supporting an average American annually) per plot, to calculate the annual impacts of a single person using each energy type (Table 2).

**Table 2:** Impacts to indicators of biodiversity and ecosystem services per unit energy^a^ produced by oil and gas or wind energy development.

Indicator (Metric)		Wind Energy	Oil & Natural Gas
1	Habitat Loss (m^2^)	247.00	105.54
3	Turbines/Reserve Pits (#)	0.02	0.01
3	Meteorological Towers/Evaporation Ponds (#)	0.0004	0.006
3	Roads (m)	9.2	4.25
3	Transmission/Power Lines (m)	0.95	0.05
4a	Linear Features (m)	15.26	7.87
4b	Temporary Disturbance (m^2^)	112.24	46.75
5	Biomass Carbon Lost (Tons of Carbon)	0.06	0.05
6a	Water Consumed (Gallons)	∼0	2,231
6b	Impervious Surfaces (m^2^)	137.00	58.95

a The unit of energy used for comparison was 317 MMBtu; roughly equivalent to the annual energy consumption of an average American [46].

We also estimated the impacts on habitat loss over 100 years to compare the short and long-term effects of each type of energy development. We explored different development scenarios which assume that attempts to restore land degraded by oil and gas development are either unsuccessful or partially (25%) successful, and that the replacement of wind turbines at the end of their life span results in some marginal increase in habitat loss (10%) or no additional habitat loss. Impact estimates are based on a 20 year reported life-span of a normal oil or natural gas well and a modern industrial scale wind turbine [45], [46].

### Data Analysis

Data recorded during the digitizing process were stored in an ArcGIS 10 file geodatabase (ESRI 2010) and statistical analysis was completed in SAS 9.3 (SAS Institute 2010). Data were evaluated on a per plot basis in one or more of the following forms for each indicator: 1) the numerical and percent change in the amount of impacts due to energy development relative to the pre-development landscape, and 2) the overall impact (from energy development plus underlying land-uses) on each indicator attributed to wind or oil and gas development. Tests for spatial correlation were non-significant for all indicators.

To compare oil and natural gas to wind energy, we used a parametric t-test to evaluate differences (alpha = 0.05) between the mean numerical and percent change in impacts for each indicator. To compare impacts in areas with energy development to areas without energy development, we used an analysis of variance which tested for differences between the three strata based on average cumulative impacts per plot. If a significant (alpha = 0.05) difference was present, we tested individual *a priori* contrasts using Bonferroni adjusted alpha levels of 0.0167 (0.05/4) per test.

## Results

We digitized a total of 6,763 unique point, line and polygon features within 375 sample plots. Our sample plots included 295 turbines (over 13% of existing wind turbines in Colorado and Wyoming) and 361 oil or gas wells on 235 well pads (approximately 0.17% of existing and historic wells in Colorado and Wyoming). The density of wells (2.9 wells/plot) and turbines (2.4 turbines/plot) in the sample plots was not significantly different (t = 1.80, df = 248, p = 0.0735). Here we 1) report results on the change in impacts to each indicator from oil and gas and wind from pre-development conditions, 2) indicate how the impacts of energy development compare to those in the reference strata and as a function of underlying land-use, 3) report impacts per unit energy consumed by the average American, and 4) estimate how these impacts change beyond the lifespan of a turbine or well.

### Direct Habitat Loss and Fragmentation

**Figure 3:**
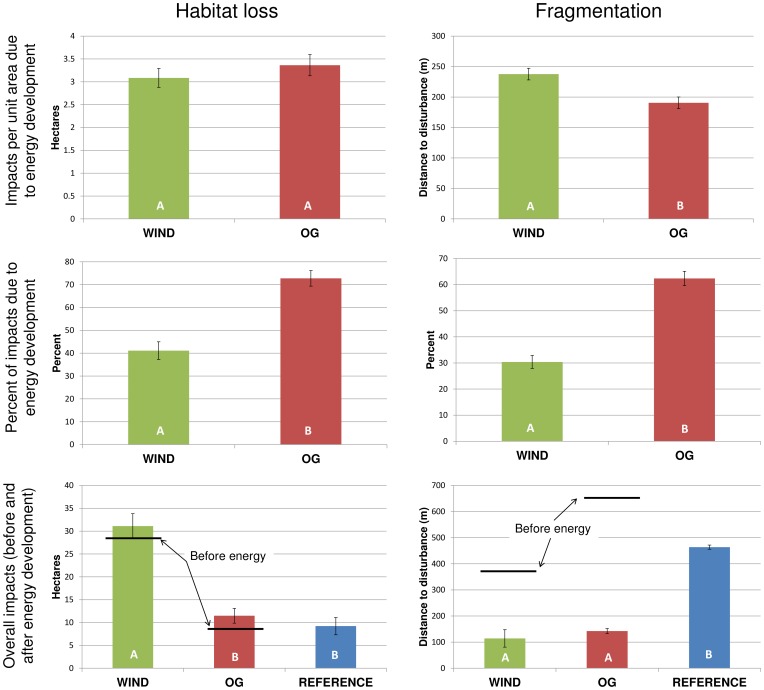
The impact of energy development on habitat loss and fragmentation. Results are presented as the impacts per unit area due to energy development (top), percent of impacts due to energy development (middle), and overall impacts from all land-uses (bottom) with horizontal black bars indicating underlying impacts prior to energy development. Different letters represent statistically significant differences (p<0.05) and error bars reflect standard errors. The y-axis on the upper and lower fragmentation graphs are distance to disturbance, therefore shorter bars represent higher levels of fragmentation.

Oil and gas and wind resulted in a similar loss of habitat per unit area, but oil and gas created a larger proportional change in habitat loss and fragmentation. The average area of direct habitat loss per plot over pre-development conditions was not significantly different between wind and oil and gas (wind = 3.09 ha, oil and gas = 3.36 ha; t = 1.18, df = 248, p = 0.239). However, considering pre-existing land-use and land cover, oil and gas accounted for 70.9% (+/– 3.47 standard error [SE]) of all habitat loss per plot, compared to just 40.3% (+/– 3.80 SE) due to wind energy. Oil and gas increased fragmentation by 62% (+/– 2.74 SE) per plot, compared to 30% (+/– 2.48 SE) from wind energy and exhibited greater fragmentation per unit area (Figure 3).

### Potential Mortality

**Figure 4:**
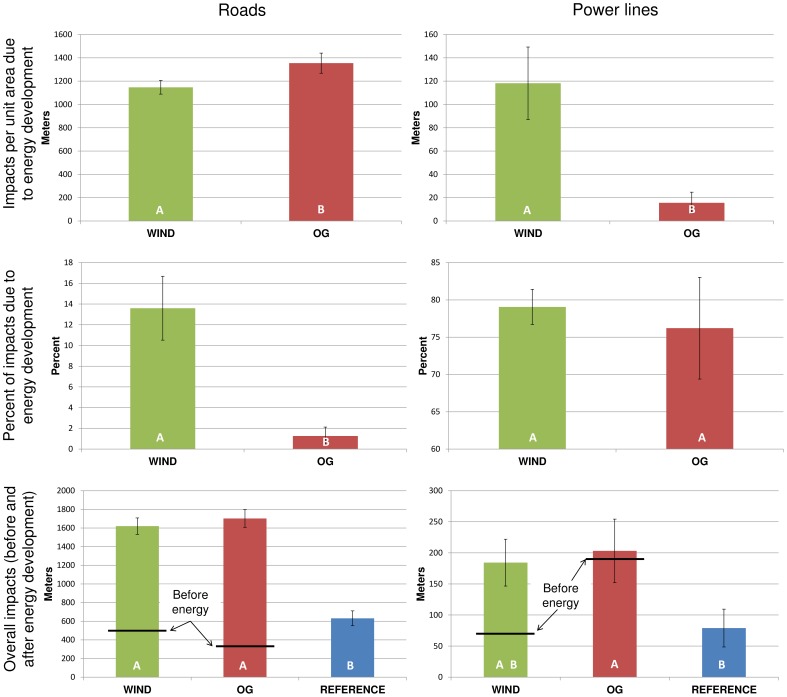
The impacts of energy development on wildlife mortality. The average length of power lines and roads is presented as the impacts per unit area due to energy development (top), percent of impacts due to energy development (middle), and overall impacts from all land-uses (bottom) with horizontal black bars indicating underlying impacts prior to energy development. Different letters represent statistically significant differences (p<0.05) and error bars reflect standard errors.

Oil and gas resulted in more meters of new road than wind energy (wind = 1,147 m, +/– 58.59 SE; oil and gas = 1,354.4 m, +/–86.14 SE), but wind energy was responsible for more new power lines (wind = 118.2 m, +/–31.08 SE; oil and gas = 15.61 m, +/–9.1 SE) per plot. Wind was responsible for a significantly greater change in meters of road than oil and gas (Figure 4). Wind energy plots averaged 2.36 (+/– 0.11 SE) wind turbines and 0.05 (+/– 0.02 SE) meteorological towers per plot. Oil and gas plots averaged 2.87 (+/– 0.27 SE) reserve pits and 0.02 (+/– 0.002 SE) evaporation ponds per plot.

### Susceptibility to Invasion

The surrogates used to assess susceptibility to biological invasion - length of linear features and area of temporary disturbance - were not significantly different between wind energy and oil and gas (linear: wind = 1,903 m, oil and gas = 2,509 m; t = 0.78, df = 248, p = 0.4361; temporary: wind = 1.40 ha, oil and gas = 1.49 ha; t = 0.27, df = 248, p = 0.7882). On average, wind energy increased the length of linear features by 1,903 m (+/– 119.84 SE), or 78.4% (+/– 2.3 SE) and increased the area of temporary disturbance by 1.4 ha (+/– 0.16 SE), or 56.3% (+/– 4.0 SE). Oil and gas, on average, increased the total length of linear features by 2,509 m (+/– 134.95 SE), or 77.7% (+/– 2.3 SE) and increased the area of temporary disturbance by 1.49 ha (+/– 0.19 SE), or 63.2% (+/– 4.1 SE).

### Carbon Stock and Water Resources

Oil and gas development was responsible for a greater loss of land-based carbon and water resources compared with wind energy. Oil and gas resulted in approximately 15.8 tons (+/– 2.98 SE) of carbon lost per plot, significantly more than wind energy (7.43 tons +/– 0.7 SE; t = 2.71, df = 248, p = 0.0071). However, taking underlying land-use and land cover into account, wind energy was responsible for 84% (+/– 2.1 SE) of all biomass carbon lost per plot, while oil and gas was only responsible for 75.5% (+/– 3.15 SE) per plot.

Oil and natural gas development, although highly variable, requires significantly greater water usage than wind energy. Current estimates indicate that crude oil, using the most common extraction technique, requires approximately 62 gallons of water per MMBtu produced, while natural gas extraction does not require water [41]. According to the COGCC [42], hydraulic fracturing, which has occurred in approximately 90% of oil and gas wells since the 1970s, uses about 1.6 million gallons of water per well (or about 80,000 gallons per year over the average 20 year life of a well). Applying these water usage estimates to the crude oil production values and type of wells in our study, an average of 711,228 gallons of water per plot is consumed from oil and gas production each year. Wind energy requires essentially no water for construction or operation [41]. The area of impervious surface resulting from energy development was not significantly different between wind and oil and gas (wind = 1.71 ha, oil and gas = 1.88 ha; t = 1.08, df = 248, p = 0.2811). However, oil and gas development contributed to an average of 63% (+/– 3.8 SE) of the impervious surface per plot, significantly more than wind energy (37.8% +/– 3.8 SE; t = 4.72, df = 248, p<0.0001).

### Comparing Energy Development to the Reference Stratum

Impacts from the combination of all human activities on our indicators were not equal across all three strata (i.e. plots with oil and gas, wind, and those lacking energy development). Habitat loss associated with wind development was significantly greater compared to plots in the oil and gas (*F*[1,372] = 44.58, p<0.0001) or reference strata (*F*[1,372] = 55.36, p<0.0001) because turbines were placed in areas with higher levels of disturbance than oil and gas (Figure 3). The pairwise comparison of oil and gas to the reference stratum was non-significant. Distance to disturbance in the reference stratum averaged 463.65 m (+/– 33.39 SE), corresponding to significantly less fragmentation than plots with wind energy (114.1 m +/– 9.85 SE; *F*[1,372] = 143.64, p<0.0001) or oil and gas (142.37 m +/– 8.0 SE; *F*[1,372] = 121.99, p<0.0001). Total fragmentation from all land-uses in the energy strata were non-significant (Figure 3).

The presence of energy development on the landscape was associated with significantly more roads than the reference stratum, and the oil and gas stratum had significantly more power lines due to all land-uses, while wind was not significantly different from the other strata (Figure 4). Considering all land-uses, the reference stratum had significantly less distance of linear features (*F*[1,372] = 61.33, p<0.0001) and significantly less area of temporary disturbance (*F*[1,372] = 12.11, p = 0.0006). The oil and gas stratum had significantly more biomass carbon loss than both the wind energy (*F*[1,372] = 7.01, p = 0.0085) and reference strata (*F*[1,372] = 6.04, p = 0.0144) when considering all land-uses. There was also no significant difference between the three strata for the total area of impervious surfaces created (*F*[2,372] = 0.29, p = 0.7454).

Underlying land cover and land-use was also dissimilar across strata. Oil and gas development occurred across a wider variety of land cover types than wind energy, including forested landscapes. Habitat loss and fragmentation (distance to disturbance) levels prior to energy development were higher in plots that contained wind energy facilities (27.97 ha +/– 2.69 SE and 264.71 m +/– 28.54 SE, respectively) compared with oil and gas fields (8.01 +/– 1.66 SE and 654.02 m +/– 36.67 SE, respectively; Figure 3) and wind energy was three times as prevalent in plots where the dominant underlying land cover was cultivated cropland (wind = 33 plots; oil and gas = 10 plots). Prior to energy development, oil and gas plots had almost 2.7 times the biomass carbon stock of the average wind energy plot (oil and gas: 912.71 tons of carbon +/– 109.47 SE; wind: 344.72 tons of carbon +/– 11.56 SE).

### Per Unit Energy and Lifespan Comparison

**Figure 5:**
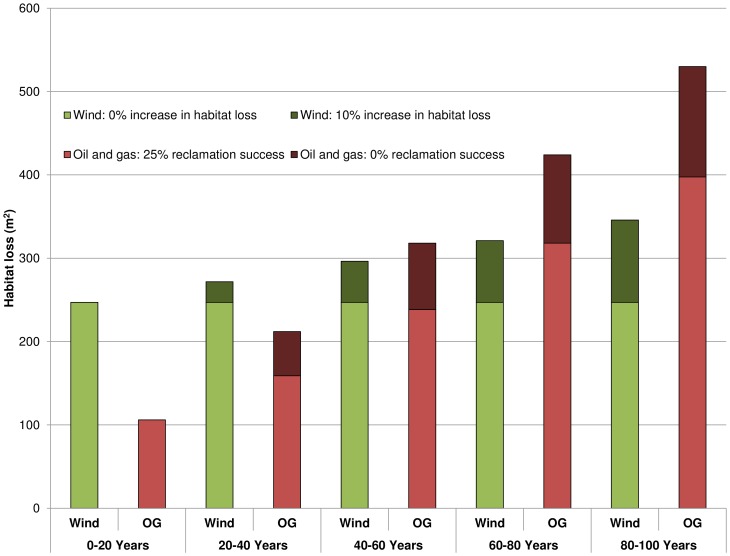
Predicted habitat loss per unit energy produced over 100 years. These trends illustrate several alternative scenarios in which: 1) there is no increase in habitat loss as wind turbines are refurbished every 20 years; 2) there is a marginal (10%) increase in habitat loss from wind energy due, for example, to repositioning turbines or widening roads; 3) there is no successful reclamation from oil and gas development; and 4) 25% of all habitat lost due to oil and gas is successfully restored every 20 years or 25% of existing infrastructure is reused. Energy production and impact estimates are based on a 20 year reported life-span of a normal oil or natural gas well and a modern industrial scale wind turbine [45], [46].

Total annual energy production per wind plot averaged 39,539 (+/– 1,800 SE) MMBtu and total energy production per oil and gas plot averaged 101,044 (+/– 20,446 SE) MMBtu. Short-term impacts per unit energy were greater due to wind energy for all indicators except water consumption. For example, within the parameters of our study an average American would require approximately 247 m^2^ (+/– 20.13 SE) of habitat loss to acquire their annual energy consumption from wind energy, but only 106 m^2^ (+/– 22.56 SE) if that energy came from oil and gas (Table 2). Habitat loss per unit energy increased substantially over 100 years of oil and gas production, exceeding the impacts per unit energy from wind. This trend held regardless of scenario (variable rates of habitat reclamation and loss associated with oil and gas and wind respectively) (Figure 5).

## Discussion

Our results demonstrate that energy development is associated with a variety of direct and indirect impacts to biodiversity and selected ecosystem services within Colorado and Wyoming. In general, oil and gas had greater net impacts to habitat loss, habitat fragmentation, carbon stock, and water resources. The susceptibility to invasion and gross habitat loss were approximately equal between the two energy types. However, underlying characteristics of the landscape, and the time period over which impacts are measured, have important implications for the nature and magnitude of energy sprawl.

**Figure 6:**
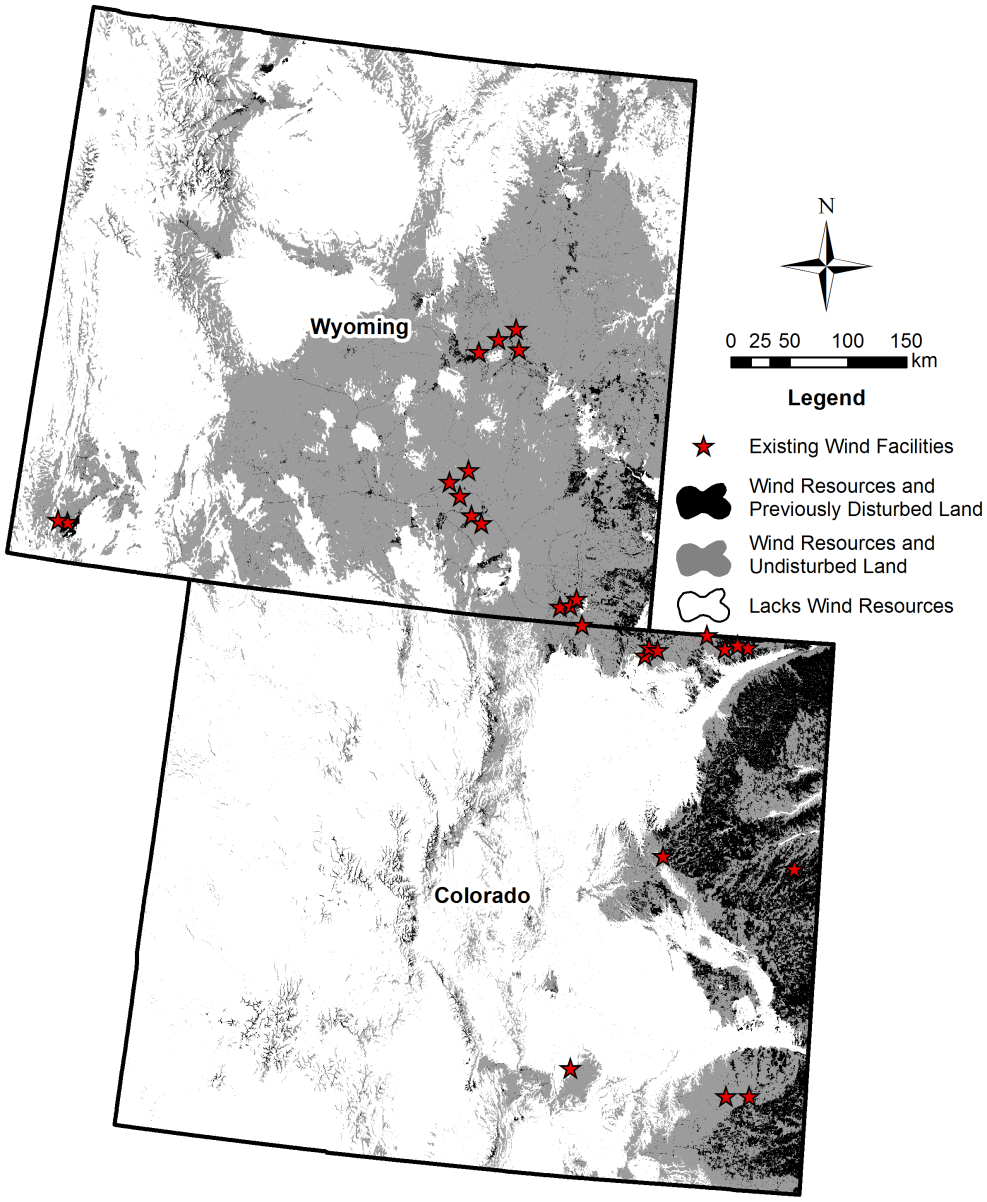
Portions of the study area where wind energy development could occur on previously disturbed lands. This map delineates areas with potential for wind energy development on previously disturbed lands (black), native or undisturbed lands (grey), and areas that lack suitable wind resources (white) as well as the location of existing wind energy facilities [27], [28], [59], [60], [61].

Comparisons of impacts across energy types must consider the geographic and landscape context. For example, regardless of energy type, development in already disturbed areas (i.e., cultivated crops) resulted in fewer impacts to indicators. Colorado and Wyoming have been proposed as two of 38 states where the DOE goals for wind energy development can be entirely met on disturbed lands (Figure 6) [47]. It remains unclear, however, if this goal is economically viable given the cost of roads, transmission lines, and other infrastructure associated with strategically developing wind resources to minimize impacts to undisturbed lands. Oil and gas developers face similar physical and economic constraints in that they can only develop in the vicinity of existing underground reserves. However, new technology which could utilize existing well pads to drill dozens of new wells has not been fully applied in Colorado and Wyoming [48]. Given the rapid pace of energy sprawl, moving quickly to establish regulations or financial incentives to develop energy resources on already disturbed land may be one of the most important steps we can take to minimize impacts to biodiversity and ecosystem services.

Similarly, the amount of biomass carbon lost due to land-use change varies with the location of development and land cover type. In this study, oil and gas development resulted in greater biomass carbon loss because a number of plots were in forested landscapes which store approximately fifteen times more biomass carbon per unit area compared with grasslands and shrub lands [19]. Additionally, because cropland generally uptakes more carbon than natural plant communities in our region (with the exception of forested landscapes), energy that replaces croplands is expected to have greater impacts on carbon sequestration compared with energy development in natural grasslands and shrublands [19].

The time frame over which energy impacts are measured substantially changes environmental outcomes. In the short-term, oil and gas development has fewer impacts per unit energy produced, but over the long-term wind energy is less detrimental. The current average life span of both an industrial scale wind turbine and an oil and gas well is approximately 20 years [45], [46]. After 20 years the wind turbine can be replaced by a new turbine on the same pad with no additional impacts. However, after the oil or gas well runs dry, a new well would need to be drilled at a new location to maintain the productivity required to meet the demands of society. Although sources of mortality will be removed, water usage will stop, and impervious surfaces will become permeable, habitat degradation from vegetation removal, fragmentation, and the presence of invasive species may continue for many years. Reclamation is often unsuccessful in a short time frame in the harsh environment of the arid west [49], and it is not clear if landscapes ever fully recover from disturbance. For this reason, per unit energy impacts to biodiversity must be considered in the context of the lifespan of the energy sources and physical structures associated with wind (renewable) and oil and gas development (finite). Through improved reclamation success and/or reuse of existing footprints, oil and gas development has the potential to reduce habitat loss over time. Similarly, to minimize habitat loss over the long-term, it is critical that the replacement of wind turbines and associated infrastructure does not substantially change the current footprint of development (Figure 5).

In the course of our analysis, we identify several substantial knowledge gaps that constrain our ability to measure the full impact of energy development. Although we were able to identify and quantify potential sources of wildlife mortality, we were unable to estimate annual mortality in our study due to a lack of reliable and empirically derived mortality data. This further highlights the need for better post-construction mortality rate estimates at energy facilities across a representative spectrum of land-use and land cover types. For example, the best available wind turbine mortality rates in the U.S. are based on only 40 existing wind facilities and represent only a small selection of ecoregions [50]. Unfortunately, mortality estimates associated with roads, power lines, and other sources are also unknown and certainly have not been collected systematically in our study area. The lack of robust mortality estimates [8] speaks to the young and largely proprietary nature of energy-wildlife research [51]. Rigorous mortality monitoring should be standard practice so that our understanding of wildlife mortality and our ability to use this information in geospatial models is based less on extrapolation and more on specific physical characteristics of the development and surrounding landscape.

Noise and light pollution are important environmental stressors that have demonstrated impacts on biodiversity and human communities [52], [53], [54]. Within active oil and gas fields, compressors and generators can reach 84 decibels [55]. Modern industrial scale wind turbines may exceed 100 decibels [56] at their loudest point; however, the noise created by turbines is tempered by the sound of the wind and dependent on the characteristics of the surrounding landscape. Additionally, vehicle traffic and temporary noise from drilling and construction contribute to noise impacts for both energy types. Artificial light sources are generally more common in wind energy facilities than oil and gas fields, where they are located at operation buildings, substations, and on some turbines. Quantifying the impacts of energy-driven noise and light pollution was not possible with available imagery and data sources, and thus was beyond the scope of our study. To accurately estimate the impacts of energy development on noise, it is critical to measure decibel levels in the field, along with the characteristics of the surrounding landscape (e.g. topography and atmospheric conditions). Noise decay and spatially explicit noise impacts (per unit area) could then be modeled using geospatial tools like SPreAD-GIS [57] and NMSim (Wyle Research & Consulting). Advancing our understanding of noise and light as byproducts of energy development should be of broad interest to decision makers concerned with the welfare of human and natural systems.

Water scarcity and contamination are global environmental challenges and the costs and benefits of energy development to water resources should not be overlooked. The water usage estimates for oil and gas wells are approximations due to widely variable characteristics of each well. However, the consumption of water as a function of energy development is based more on characteristics of the industry rather than the landscape; wind energy does not require water, regardless of location. Therefore, measures to address water use should be focused on the specific actions of the oil and gas industry and location of water sources. For example, hydraulic fracturing can open new natural gas supplies, but the process requires large amounts of water and uses chemicals that could result in negative impacts on water quality [58].

The six indicators analyzed in this study are important to wildlife conservation and human well-being; however, they are not equivalent for all taxa or human communities. It is not clear, for example, how the net losses associated with direct mortality compare to the indirect population-level impacts of habitat loss. Additionally, we emphasize that an analysis of the full impacts of energy development on indicators of biodiversity and ecosystem services must address the effects of long-distance transport, processing and the use of these different forms of energy. There is good reason to believe that the land-use required for transmission (roads, pipelines, power lines) has and will continue to have important and wide-ranging impacts on wildlife habitat and human well-being [6]. We focused on the farm or field where production occurred because the local and regional “footprint” of energy development has not been fully appreciated. However, others have studied the life-cycle impacts of energy development [6], [45] and we strongly encourage policy makers, developers and others involved in energy decision making to consider the effects of both regional and site-level impacts on biodiversity and human well-being.

Although this study is a retrospective analysis of land-use in the intermountain west, the implications of this work go far beyond this region. This approach could be used to predict future impacts on particular species or ecosystems or to define the nature and quantify the magnitude of mitigation needed to counter these impacts. For example, there is substantial interest in understanding the cumulative impacts of development on particularly sensitive species such as Greater Sage-Grouse (*Centrocercus urophasianus*), sage-brush obligate songbirds, or Golden Eagles (*Aquila chrysaetos*). Our approach and findings can also be used pro-actively to site wind or oil and gas development in places where it will have minimal impacts on the loss of natural ecosystems, the storage and sequestration of carbon for climate mitigation, and the sustainability of other land-based natural capital.

Demonstrating how energy use by an average consumer impacts the landscape could be valuable for empowering citizens to make informed decisions and take meaningful actions. By highlighting the area of potential wildlife habitat lost as a result of energy development (or sustained from energy savings), we have the potential to influence people’s daily decisions in a world where information on the true environmental and social costs of our behavior is often elusive. Ultimately, our integrative approach and our specific findings can be used to help address one of the most daunting challenges facing society: balancing the demand for energy with the desire to conserve land for the biodiversity and ecosystem services we value.
